# Improvement of motor function in mice after implantation of mononuclear stem cells from human umbilical cord and placenta blood after 3 and 6 weeks of experimental spinal cord injury

**DOI:** 10.1016/j.clinsp.2024.100509

**Published:** 2024-10-10

**Authors:** Thiego Pedro Freitas Araujo, Alexandre Fogaça Cristante, Raphael Martus Marcon, Gustavo Bispo dos Santos, Maria Helena Alves Nicola, Alex Oliveira de Araujo, Fernando Barbosa Sanchez, Tarcísio Eloy Pessoa de Barros Filho

**Affiliations:** a Department of Orthopedic Surgery, Hospital Sírio Libanês, Brasília, DF, Brazil; b Department of Orthopedic Surgery, Instituto de Ortopedia e Traumatologia da Universidade de São Paulo, São Paulo, SP, Brazil; c Cryopraxis Cryobiology, Espírito Santo do Pinhal, SP, Brazil; d Department of Orthopedic Surgery, Rede SARAH de Hospitais de Reabilitação, SMHS - Área Especial, Brasília, DF, Brazil

**Keywords:** Mesenchymal stem cells, Spinal cord trauma, Umbilical cord, Motor evaluation, Histopathological evaluation

## Abstract

**Study design:**

Experimental study utilizing with a standardized model (MASCIS Impactor) of Spinal Cord Injury (SCI) in Balb C mouse model with implantation of mononuclear stem cells derived from the human umbilical cord and placenta blood in the early chronic phase of SCI.

**Objectives:**

The aim of this study was to evaluate the nerve regeneration and motor functional recovery in Balb C mice with surgically induced paraplegia in response to the use of mononuclear stem cells, in early chronic phase (> 2 weeks and < 6 months), because there is yet potential of neuronal and functional recovery as the neuronal scar is not still completely established.

**Methods:**

Forty-eight mice were randomly assigned to 6 groups of 8 animals. Group 1 received the stem cells 3 weeks after the trauma, and Group 2 received them six weeks later. In Group 3, saline solution was injected at the site of the lesion 3 weeks after the trauma, and in Group 4, 6 weeks later. Group 5 underwent only spinal cord injury and Group 6 underwent laminectomy only. The scales used for motor assessment were BMS and MFS for 12 weeks.

**Results:**

The intervention groups showed statistically significant motor improvement. In the histopathological analysis, the intervention groups had a lower degree of injury (p < 0.05). Regarding axonal budding, the intervention groups showed increasing in axonal budding in the caudal portion (p < 0.05).

**Conclusions:**

The use of stem cells in mice in the chronic phase after 3 and 6 weeks of SCI brings functional and histopathological benefits to them.

## Highlights

• The chronic stage of spinal cord injury is the less studied group in animal models.
• The biggest pool of patients with spinal cord injury are in a chronic stage.
• Human umbilical cord and placental blood are highly available and can be used allogeneically.
• Mice showed improved motor function after implantation of stem cells in the chronic stage.
• Better function was achieved when stem cells were implanted near the subacute phase (3 weeks > 6 weeks).

## Introduction

Spinal cord injury is one of the great challenges of medicine. It constitutes catastrophic damage for the affected patients and no measure to date has been shown to be frankly effective for the treatment, which remains a challenge.^1^^,^^2^ The alterations resulting from spinal cord injury are divided into acute, subacute, and chronic. Acute injury leads to secondary injury, widening the neurological injury. Finally, the chronic phase leads to the progression of neurological dysfunction, both anterograde and retrograde, which can affect brain regions.^3^

Stem cells are cell clones capable of self-renewal and multilineage differentiation.^4^ Due to their ability to differentiate into any cell, they are a great hope for the treatment of degenerative diseases in general, especially those with neurological damage, such as stroke and MRT.

Umbilical cord and human placenta mesenchymal and mononuclear stem cells have a great capacity to be used allogeneically, including as xenografts. This ability is due to the inability of these cells to act as Alloantigen Presenting Cells (APCs) or antigens themselves, in addition to inhibiting the lymphocyte response against allogens. This characteristic derives from secondary soluble factors of the interaction between stem cells and peripheral blood mononuclear allogeneic cells.^5^

In the literature, there are several experimental studies of stem cells in animals in the acute and sub-acute phases and fewer studies on the use of stem cells in the chronic phase.^6^, ^7^, ^8^, ^9^ Studying this third period of the injury is more challenging because it covers a wide period of time since a 3-week injury and a one-year injury are both in the same group. The authors see studies with implantation of stem cells at 28 weeks showing motor improvement,^6^ while in other studies with implantation at 3 months^8^ or 10 months^9^ of injury, despite showing histological improvement, they did not show any motor benefit for the animals studied. Due to the large time span that defines the chronic phase, it is important to study different periods of this phase, in order to understand the time limit of the lesion for the use of stem cells and to be able to offer, in the future, in the appropriate time, to the population of chronic spinal cord injuries who may benefit from the treatment.

There are authors in the literature who classify the period between 2 weeks and 6 months as an intermediate phase, a phase between the subacute and chronic phases.^10^ In this phase, the maturation of the glial scar continues. In addition, the death of oligodendrocytes leads to loss of axonal conduction potential and loss of function, secondary to axonal demyelination.^11^ There is evidence that remyelination occurs in the intermediate phase, however in an anomalous way, being very thin, preventing the proper functioning of nerve impulse conduction.^12^ Considering that there is potential for histopathological and clinical improvement in this initial chronic phase or intermediate phase, the time of stem cell injection between 3 and 6 weeks was chosen.

In a recent review of the literature, it was observed that 1 % of the studies study the intermediate phase of spinal cord injury in animals. In humans, the vast majority of studies are done in the chronic phase (83 %).^10^ The importance of this study is to shed light on a phase in which there is still greater potential for neurological recovery so that it can bring useful information for scientific improvement in order to be used, in the future, in humans.

As spinal cord injury still is a great burden to patients and society and none of the actual treatments are a game changer in patients’ lives, the stem cells therapy has become a great source of hope. As there is a lack of knowledge in treatment with stem cells in the early chronic phase of spinal cord injury, both in animals and humans, and most part of humans patients are in the chronic phase, the authors designed this study to clarify if there is improvement in motor function and histologically, and also if there is different results in different time lapses of intervention.

The main objective of the study is to evaluate the motor functional recovery and neuronal regeneration in Balb C mice, among randomized groups, after surgically provoked spinal cord injury followed by injection of mononuclear stem cells from human umbilical cord and placenta blood applied after 3 weeks and 6 weeks of injury, in the chronic phase of neurological recovery.

## Materials and methods

### Ethics

The research protocol was submitted to and approved by the Scientific Committee of the Institute of Orthopedics and Traumatology of HC-FMUSP and by the Ethics Committee for Analysis of Research Projects (CAPPesp) of the Hospital das Clínicas of the Faculty of Medicine of the University of São Paulo (HCFMUSP) through the Ethics Committee on the Use of Animals in protocol 1248/2019. All research is being conducted ethically according to the standards established by the ARRIVE guidelines and was conducted in accordance with the National Research Council's Guide for the Care and Use of Laboratory Animals, the Canadian Council on Animal Care and the Brazilian College of Animal Experimentation.

### Of the animals and inclusion and exclusion criteria

A total of 48 Balb C mice were randomly allocated to groups, juveniles with average age between 10 and 12 weeks, males, weighing around 70 to 100 g and were divided into 6 groups of 8 animals. The authors used one cage (40 × 60 cm) for every three mice in the same litter. The cages were kept in an air-conditioned environment, in good hygienic conditions, and the mice were provided with adequate food and hydration at the Laboratory of Spinal and Nerve Trauma Studies of the Institute of Orthopedics and Traumatology of HC-FMUSP. Cases of death after experimental spinal cord injury were excluded; abnormalities in the injured area of the spinal cord observed macroscopically; autophagy or mutilation among animals; normal movement in the first evaluation after injury; Complete paraplegia seen after the initial injury.

### Collection and processing of umbilical cord and placental blood

The human umbilical cord and placenta blood was collected from newborns whose guardians agreed to participate in the study, after signing the informed consent form, meeting the criteria required by CNS Resolution n° 466 of 2012, at the Gynecology and Obstetrics Unit of the Hospital das Clínicas from University of São Paulo. The company Cryopraxis® Criobiologia Ltda, Cell Processing Center, was responsible for all stages of material handling, and proceeded according to appropriate technical protocols, obeying the criteria of good national and international practices.

### Of the experimental groups

48 animals were divided into 6 groups with 8 animals:

Group 1: The animals were submitted to the spinal cord contusion due to a fall in weight (10g) of 12.5 mm height, using the MASCIS Impactor model. A volume of 4 × 10^5^/40 μL was applied to the lesion site of mononuclear stem cells from human umbilical cord and placenta blood as a single dose at epidural space, 3 weeks after spinal cord contusion.
Group 2: The animals were submitted to the spinal cord contusion due to a fall in weight (10g) of 12.5 mm height, using the MASCIS Impactor model. A volume of 4 × 10^5^/40 μL was applied to the lesion site of mononuclear stem cells from human umbilical cord and placenta blood as a single dose at epidural space, 6 weeks after spinal cord contusion.
Group 3: The animals were submitted to the spinal cord contusion due to a fall in weight (10g) of 12.5 mm height, using the MASCIS Impactor model. A single dose of 0.9 % saline solution was applied to the lesion site at epidural space 3 weeks after spinal cord contusion.
Group 4: The animals were submitted to the spinal cord contusion due to a fall in weight (10g) of 12.5 mm height, using the MASCIS Impactor model. A single dose of 0.9 % saline solution was applied to the lesion site at the epidural spance 6 weeks after spinal cord contusion.
Group 5: The animals were submitted to the spinal cord contusion due to a fall in weight (10g) of 12.5 mm height, using the MASCIS Impactor model.
Group 6: The animals were patients who underwent access and laminectomy without spinal cord injury.

After confirmation of the anesthetic plane by tightening the hind leg, trichotomy was performed in the dorsal region, antisepsis with chlorhexidine, and longitudinal incision in the topography from T7 to T11. After dissection by planes and exposure of the paravertebral muscles, the posterior components of the dorsal-lumbar spine were exposed. Subsequently, a T8 laminectomy was performed in the caudocranial direction for spinal cord exposure. The lesions were produced according to the international protocol MASCIS (Multicenter Animal Spinal Cord Injury Study);^13^ the NYU Impactor system,^14^^,^^15^ The injured site was irrigated with saline solution at room temperature, followed by an inspection, cauterization, tissue approximation and skin suturing.

### Functional evaluation

The motor evaluation of the mice was performed during the post-injury period, according to the following schedule:

- Basso Mouse Scale (BMS) on the 2^nd^, 7^th^, 14^th^, 21^st^, 28^th^, 35^th^, 42^nd^, 49^th^, 56^th^, 63^rd^, 70^th^, 77^th^, and 84^th^ day after injury.^16^
- Motor Function Score (MFS) on the 2^nd^, 7^th^, 14^th^, 21^st^, 28^th^, 35^th^, 42^nd^, 49^th^, 56^th^, 63^rd^, 70^th^, 77^th^ and 84^th^ day after injury.^17^

### Histopathological evaluation

The anatomopathological study consisted of microscopic (optical) analysis of slides stained with Hematoxylin-Eosin (HE). Histological sections were made in the axial plane of the medullary segment at intervals of two millimeters, representing the entire injured area, in an extension of one centimeter from the center of the lesion. The classifications that were used in the microscopic evaluation of the spinal cords will be the following items: necrosis, hemorrhage, hyperemia, degenerate and cellular infiltrate. The criteria will be scored according to the following score: absent, discrete, moderate, and intense.

### Axonal budding

Myelinated fibers were counted in the cranial segments, lesions and caudal segments of the spinal cord, using sigma scan pro software.

### Statistical analysis

The parameters evaluated were described according to groups and evaluation moments with the use of summary measures (mean, standard deviation, median, minimum and maximum) and the parameters were compared using Generalized Estimation Equations (GEE) with normal distribution and identity linkage function, assuming a matrix of 1^st^ order autoregressive correlations between the evaluation moments for the BMS and MFS using the lowest value obtained on both sides was used for the analyses.^18^ All analyses were followed by multiple Bonferroni comparisons to identify between which groups or moments the differences occurred,^19^ while the histological parameters were described according to groups using absolute and relative frequencies and compared between groups using Kruskal-Wallis tests followed by Dunn's multiple comparisons when the differences occurred.^19^ Quantitative histological parameters were described according to groups using summary measures and compared using analysis of variance (ANOVA) followed by multiple Bonferroni comparisons if differences were found. To perform the analyses, the software IBM-SPSS for Windows version 22.0 and the software Microsoft Excel 2013. The authors started from the null hypothesis of equity, considering the probability of error of type I of 5 % and type II of 20 %.

## Results

After conducting the experiments in the groups of the six groups of mice, the authors obtained the results below.

Table 1 shows that the mean behavior of the groups over the moments evaluated for both scales was statistically different (p _Interaction_ < 0.001).

**Table 1:** Description of the BMS and MFS scales according to groups and moments of evaluation and results of comparisons.

Variable/Group	Period of evaluation								p Group	p Moment	p Interaction
	Before	2 Days	2 weeks	4 weeks	6 weeks	8 weeks	10 weeks	12 weeks			
**BMS**									**<0.001**	**<0.001**	**<0.001**
**Contusion + 1 dose 3w**											
Mean ± SD	9 ± 0	0 ± 0	0.63 ± 0.74	1.38 ± 0.52	2.5 ± 0.54	2.75 ± 0.71	3.25 ± 0.46	3.88 ± 0.64			
Median (min.; max.)	9 (9; 9)	0 (0; 0)	0.5 (0; 2)	1 (1; 2)	2.5 (2; 3)	3 (2; 4)	3 (3; 4)	4 (3; 5)			
**Contusion + 1 dose 6w**											
Mean ± SD	9 ± 0	0 ± 0	0.38 ± 0.52	2.25 ± 0.71	2.25 ± 0.71	2.75 ± 0.46	3 ± 0	3.38 ± 0.52			
Median (min.; max.)	9 (9; 9)	0 (0; 0)	0 (0; 1)	2 (1; 3)	2 (1; 3)	3 (2; 3)	3 (3; 3)	3 (3; 4)			
**Contusion + Saline 3w**											
Mean ± SD	9±0	0±0	0.5±0.54	1.38±0.52	1.88±0.35	1.88±0.35	1.5±0.54	1.63±0.52			
Median (min.; max.)	9 (9; 9)	0 (0; 0)	0.5 (0; 1)	1 (1; 2)	2 (1; 2)	2 (1; 2)	1.5 (1; 2)	2 (1; 2)			
**Contusion + Saline 6w**											
Mean ± SD	8.88 ± 0.35	0 ± 0	0 ± 0	2 ± 0	2 ± 0.54	2 ± 0.54	2.13 ± 0.64	2.63 ± 0.52			
Median (min.; max.)	9 (8; 9)	0 (0; 0)	0 (0; 0)	2 (2; 2)	2 (1; 3)	2 (1; 3)	2 (1; 3)	3 (2; 3)			
**Contusion**											
Mean ± SD	9 ± 0	0 ± 0	0.63 ± 0.52	1.5 ± 0.54	2 ± 0.54	2.13 ± 0.64	1.88 ± 0.64	1.88 ± 0.64			
Median (min.; max.)	9 (9; 9)	0 (0; 0)	1 (0; 1)	1,5 (1; 2)	2 (1; 3)	2 (1; 3)	2 (1; 3)	2 (1; 3)			
**Sham**											
Mean ± SD	9 ± 0	9 ± 0	9 ± 0	9 ± 0	9 ± 0	9 ± 0	9 ± 0	9 ± 0			
Median (min.; max.)	9 (9; 9)	9 (9; 9)	9 (9; 9)	9 (9; 9)	9 (9; 9)	9 (9; 9)	9 (9; 9)	9 (9; 9)			
**MFS**									**<0.001**	**<0.001**	**<0.001**
**Contusion + 1 dose 3w**											
Mean ± SD	11.5 ± 0.93	0 ± 0	0.5 ± 0.54	1.5 ± 0.54	2.75 ± 1.39	3.25 ± 1.39	4±1.31	4.75 ± 0.89			
Median (min.; max.)	11.5 (10; 13)	0 (0; 0)	0.5 (0; 1)	1.5 (1; 2)	3 (1; 4)	4 (1; 5)	4 (1; 5)	4.5 (4; 6)			
Contusion + 1 dose 6w											
Mean ± SD	11.38 ± 1.19	0 ± 0	0.38 ± 0.52	1.75 ± 0.46	3.25 ± 1.04	3.5 ± 0.93	3.75 ± 0.71	4.13 ± 0.99			
Median (min.; max.)	11 (10; 13)	0 (0; 0)	0 (0; 1)	2 (1; 2)	4 (2; 4)	4 (2; 4)	4 (2; 4)	4 (2; 5)			
Contusion + Saline 3w											
Mean ± SD	10.88 ± 0.83	0 ± 0	0.5 ± 0.54	1.25 ± 0.46	1.5 ± 0.54	1.75 ± 0.46	1.38 ± 0.52	1.5 ± 0.54			
Median (min.; max.)	11 (10; 12)	0 (0; 0)	0.5 (0; 1)	1 (1; 2)	1.5 (1; 2)	2 (1; 2)	1 (1; 2)	1.5 (1; 2)			
Contusion + Saline 6w											
Mean ± SD	11.63 ± 0.92	0 ± 0	0 ± 0	1.75 ± 0.46	2.38 ± 1.06	2.13 ± 0.84	2.88 ± 1.25	2.75 ± 1.04			
Median (min.; max.)	11 (11; 13)	0 (0; 0)	0 (0; 0)	2 (1; 2)	2 (1; 4)	2 (1; 4)	3 (1; 4)	2 (2; 4)			
Contusion											
Mean ± SD	11.75 ± 0.89	0 ± 0	0.63 ± 0.52	1.5 ± 0.54	1.88 ± 0.35	1.88 ± 0.35	1.75 ± 0.46	2.13 ± 0.84			
Median (min.; max.)	11.5 (11; 13)	0 (0; 0)	1 (0; 1)	1.5 (1; 2)	2 (1; 2)	2 (1; 2)	2 (1; 2)	2 (1; 4)			
Sham											
Mean ± SD	11.5 ± 0.53	11.63 ± 0.52	11.5 ± 0.54	11.5 ± 0.54	11.5 ± 0.54	11.5 ± 0.54	11.38 ± 0.74	11.5 ± 0.54			
Median (min.; max.)	11.5 (11; 12)	12 (11; 12)	11.5 (11; 12)	11.5 (11; 12)	11.5 (11; 12)	11.5 (11; 12)	11.5 (10; 12)	11.5 (11; 12)			

GEE with normal distribution and identity link function, assuming AR(1) correlation matrix between moments; Only moments 2, 6 and 12 weeks were used for analysis and the Sham group was not used.

**Fig. 1:**
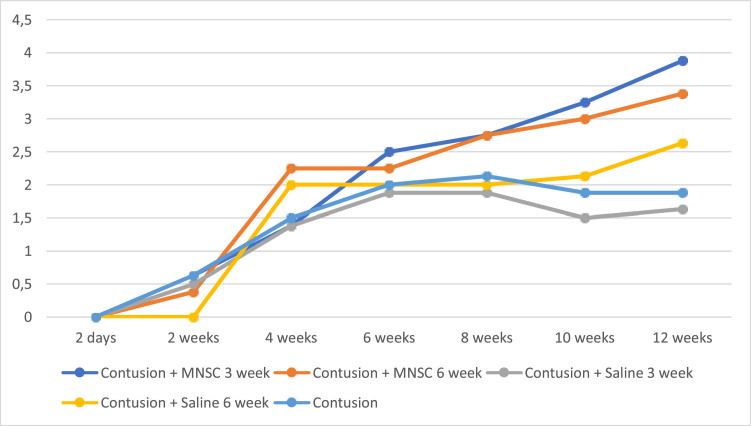
Evolution of BMS by week. MNSC, Mononuclear Stem Cells.

In the Fig. 1, the authors see the evolution of the groups of mice per week on the BMS scale. All groups in the pre-injury period had a value of 9, as well as Group 6 (Sham) had this value on the BMS scale throughout the experiment. For the purpose of interpretation and analysis of the Fig., the pre-injury time and Group 6 were removed from it.

Table 2 shows that the BMS scale of the treated groups actually increased on a statistically average at each moment of evaluation, whereas in the groups in which saline or untreated solution was used, there was a statistically significant mean increase from 2 weeks to the other weeks (p < 0.05), but there was no statistically significant mean difference from 6 to 12 weeks (p > 0.05). Only at 12 weeks there was a statistically significant mean difference between the groups, mainly between the effectively treated groups and the groups in which serum was used or not treated (p < 0.05).

**Table 2:** Results of multiple comparisons of the BMS scale between groups and evaluation moments.

BMS						
Group/Time	Comparison	Mean difference	Standard Error	p	95 % CI	
					Inferior	Superior
Contusion + 3-week M NSC	2 weeks vs. 6 weeks	-1.88	0.28	**<0.001**	-2.84	-0.91
	2 weeks vs. 12 weeks	-3.25	0.27	**<0.001**	-4.21	-2.30
	6 weeks vs. 12 weeks	-1.38	0.28	**<0.001**	-2.34	-0.41
Contusion + 6-week M NSC	2 weeks vs. 6 weeks	-1.88	0.28	**<0.001**	-2.84	-0.91
	2 weeks vs. 12 weeks	-3.00	0.27	**<0.001**	-3.96	-2.05
	6 weeks vs. 12 weeks	-1.13	0.28	**0.005**	-2.09	-0.16
Contusion + 3-week saline	2 weeks vs. 6 weeks	1.38	0.28	**<0.001**	-2.34	-0.41
	2 weeks vs. 12 weeks	-1.13	0.27	**0.004**	-2.08	-0.17
	6 weeks vs. 12 weeks	0.25	0.28	>0.999	-0.71	1.21
Contusion + 6-week saline	2 weeks vs. 6 weeks	-2.00	0.28	**<0.001**	-2.96	-1.04
	2 weeks vs. 12 weeks	-2.63	0.27	**<0.001**	-3.58	-1.67
	6 weeks vs. 12 weeks	-0 63	028	>0.999	-1.59	0.34
Contusion	2 weeks vs. 6 weeks	-1.38	0.28	<0.001	-2.34	-0.41
	2 weeks vs. 12 weeks	1.25	0.27	0.001	-2.21	-0.30
	6 weeks vs. 12 weeks	0.13	0.28	>0.999	-0.84	1.09
2 weeks	Contuson + 3w MNSC vs. Contuson + 6w M NSC	0.25	0.27	>0.999	-0.71	1.21
	Contuson + 3w MNSC vs. Contuson + 3w saline	0.13	0.27	>0.999	-0.83	1.08
	Contuson + 3w MNSC vs. Contuson + 6w saline	0.63	0.27	>0.999	-0.33	1.58
	Contuson + 3w MNSC vs. Contuson	0.00	0.27	>0.999	-0.96	0.96
	Contuson + 6w MNSC vs. Contuson + 3w saline	-0.13	0.27	>0.999	1.08	0.83
	Contuson + 6w MNSC vs. Contuson + 6w saline	0.38	0.27	>0.999	-0.58	1 33
	Contuson + 6w MNSC vs. Contuson	-0.25	0.27	>0.999	-1.21	0.71
	Contuson + 3w MNSC vs. Contuson + 6w saline	0.50	0.27	>0.999	-0.46	1.46
	Contuson + 3w MNSC vs. Contuson	-0.13	0.27	>0.999	1 08	0.83
	Contuson + 6w MNSC vs. Contuson	-0.63	0.27	>0.999	-1.58	0.33
6 weeks	Contuson + 3w MNSC vs. Contuson + 6w M NSC	0.25	0.27	>0.999	-0.71	1.21
	Contuson + 3w MNSC vs. Contuson + 3w saline	0.63	0.27	>0.999	-0.33	1.58
	Contuson + 3w MNSC vs. Contuson + 6w saline	0.50	0.27	>0.999	-0.46	1.46
	Contuson + 3w MNSC vs. Contuson	0.50	0.27	>0.999	-0.46	1.46
	Contuson + 6w MNSC vs. Contuson + 3w saline	0.38	0.27	>0.999	-0.58	1.33
	Contuson + 6w MNSC vs. Contuson + 6w saline	0.25	0.27	>0.999	-0.71	1.21
	Contuson + 6w MNSC vs. Contuson	0.25	0.27	>0.999	-0.71	1.21
	Contuson + 3w MNSC vs. Contuson + 6w saline	-0.13	0.27	>0.999	-1.08	0.83
	Contuson + 3w MNSC vs. Contuson	-0.13	0.27	>0.999	-1.08	0.83
	Contuson + 6w MNSC vs. Contuson	0.00	0.27	>0.999	-0.96	0.96
12 weeks	Contuson + 3w MNSC vs. Contuson + 6w M NSC	0.50	0.27	>0.999	-0.46	1.46
	Contuson + 3w MNSC vs. Contuson + 3w saline	2.25	0.27	**<0.001**	1.30	3.21
	Contuson + 3w MNSC vs. Contuson + 6w saline	1.25	0.27	**0.001**	0.30	2.21
	Contuson + 3w MNSC vs. Contuson	2.00	0.27	**<0.001**	1.05	2.96
	Contuson + 6w MNSC vs. Contuson + 3w saline	1.75	0.27	**<0.001**	0.80	2.71
	Contuson + 6w MNSC vs. Contuson + 6w saline	0.75	0.27	0.637	-0.21	1.71
	Contuson + 6w MNSC vs. Contuson	1.50	0.27	**<0.001**	0.55	2.46
	Contuson + 3w MNSC vs. Contuson + 6w saline	-1.00	0.27	**0.027**	-1.96	-0.05
	Contuson + 3w MNSC vs. Contuson	-0.25	0.27	>0.999	-1.21	0.71
	Contuson + 6w MNSC vs. Contuson	0.75	0.27	0.637	-0.21	1.71

**Fig. 2:**
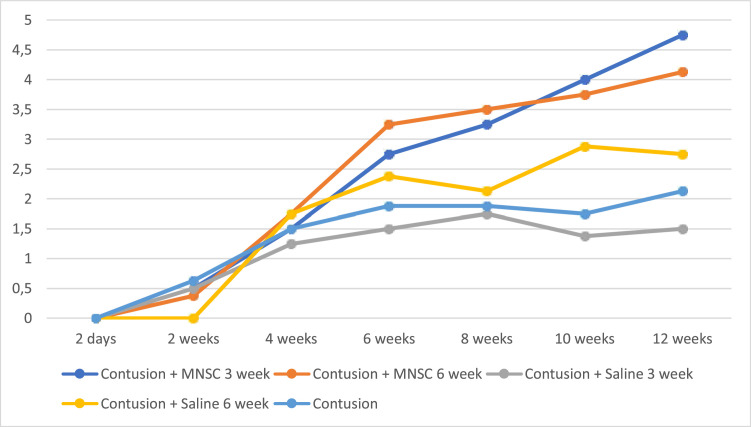
Evolution of MFS by week. MNSC, Mononuclear Stem Cells.

In Fig. 2, the authors see the evolution of the groups of mice per week on the MFS scale. All pre-injury groups had a normal value close to 11, as well as Group 6 (Sham) had this value on the MFS scale throughout the experiment. For the purpose of interpretation and analysis of the Fig., the pre-injury time and Group 6 were removed from it.

Table 3 shows that the group treated effectively earlier showed statistically significant mean improvement in the scale at each moment of evaluation (p < 0.05), while the group treated effectively later showed a mean increase in the scale of only 2 weeks for the others (p < 0.05), as well as the groups treated with serum after 6 weeks and the untreated group. Only at 12 weeks there were statistically significant mean differences between the groups, with mainly the effectively treated groups having statistically higher mean values than the groups in which serum or untreated was used (p < 0.05).

**Table 3:** Results of multiple comparisons of the MFS scale between groups and evaluation moments.

BMS						
Group/Time	Comparison	Mean difference	Standard Error	p	95 % CI	
					Inferior	Superior
Contusion + 3-week M NSC	2 weeks vs. 6 weeks	-2.25	0.35	**<0.001**	-3.46	-1.04
	2 weeks vs. 12 weeks	-4.25	0.39	**<0.001**	-5.60	-2.90
	6 weeks vs. 12 weeks	-2.00	0.35	**<0.001**	-3.21	-0.79
Contusion + 6-week M NSC	2 weeks vs. 6 weeks	-2.88	0.35	**<0.001**	-4.08	-1.67
	2 weeks vs. 12 weeks	-3.75	0.39	**<0.001**	-5.10	-2.40
	6 weeks vs. 12 weeks	-0.88	0.35	>0.999	-2.08	0.33
Contusion + 3-week saline	2 weeks vs. 6 weeks	-1.00	0.35	0.397	-2.21	0.21
	2 weeks vs. 12 weeks	-1.00	0.39	0.990	-2.35	0.35
	6 weeks vs. 12 weeks	0.00	0.35	>0.999	-1.21	1.21
Contusion + 6-week saline	2 weeks vs. 6 weeks	-2.38	0.35	**<0.001**	-3.58	-1.17
	2 weeks vs. 12 weeks	-2.75	0.39	**<0.001**	-4.10	-1.40
	6 weeks vs. 12 weeks	-0.38	0.35	>0.999	-1.58	0.83
Contusion	2 weeks vs. 6 weeks	-1.25	0.35	**0.031**	-2.46	-0.04
	2 weeks vs. 12 weeks	-1.50	0.39	**0.010**	-2.85	-0.15
	6 weeks vs. 12 weeks	-0.25	0.35	>0.999	-1.46	0.96
2 weeks	Contuson + 3w MNSC vs. Contuson + 6w M NSC	0.13	0.40	>0.999	-1.26	1.51
	Contuson + 3w MNSC vs. Contuson + 3w saline	0.00	0.40	>0.999	-1.39	1.39
	Contuson + 3w MNSC vs. Contuson + 6w saline	0.50	0.40	>0.999	-0.89	1.89
	Contuson + 3w MNSC vs. Contuson	-0.13	0.40	>0.999	-1.51	1.26
	Contuson + 6w MNSC vs. Contuson + 3w saline	-0.13	0.40	>0.999	-1.51	1.26
	Contuson + 6w MNSC vs. Contuson + 6w saline	0.38	0.40	>0.999	-1.01	1.76
	Contuson + 6w MNSC vs. Contuson	-0.25	0.40	>0.999	-1.64	1.14
	Contuson + 3w MNSC vs. Contuson + 6w saline	0.50	0.40	>0.999	-0.89	1.89
	Contuson + 3w MNSC vs. Contuson	-0.13	0.40	>0.999	-1.51	1.26
	Contuson + 6w MNSC vs. Contuson	-0.63	0.40	>0.999	-2.01	0.76
6 weeks	Contuson + 3w MNSC vs. Contuson + 6w M NSC	-0.50	0.40	>0.999	-1.89	0.89
	Contuson + 3w MNSC vs. Contuson + 3w saline	1.25	0.40	0.173	-0.14	2.64
	Contuson + 3w MNSC vs. Contuson + 6w saline	0.38	0.40	>0.999	-1.01	1.76
	Contuson + 3w MNSC vs. Contuson	0.88	0.40	>0.999	-0.51	2.26
	Contuson + 6w MNSC vs. Contuson + 3w saline	1.75	0.40	**0.001**	0.36	3.14
	Contuson + 6w MNSC vs. Contuson + 6w saline	0.88	0.40	>0.999	-0.51	2.26
	Contuson + 6w MNSC vs. Contuson	1.38	0.40	0.056	-0.01	2.76
	Contuson + 3w MNSC vs. Contuson + 6w saline	-0.88	0.40	>0.999	-2.26	0.51
	Contuson + 3w MNSC vs. Contuson	-0.38	0.40	>0.999	-1.76	1.01
	Contuson + 6w MNSC vs. Contuson	0.50	0.40	>0.999	-0.89	1.89
12 weeks	Contuson + 3w MNSC vs. Contuson + 6w M NSC	0.63	0.40	>0.999	-0.76	2.01
	Contuson + 3w MNSC vs. Contuson + 3w saline	3.25	0.40	<0.001	1.86	4.64
	Contuson + 3w MNSC vs. Contuson + 6w saline	2.00	0.40	<0.001	0.61	3.39
	Contuson + 3w MNSC vs. Contuson	2.63	0.40	<0.001	1.24	4.01
	Contuson + 6w MNSC vs. Contuson + 3w saline	2.63	0.40	<0.001	1.24	4.01
	Contuson + 6w MNSC vs. Contuson + 6w saline	1.38	0.40	0.056	-0.01	2.76
	Contuson + 6w MNSC vs. Contuson	2.00	0.40	<0.001	0.61	3.39
	Contuson + 3w MNSC vs. Contuson + 6w saline	-1.25	0.40	0.173	-2.64	0.14
	Contuson + 3w MNSC vs. Contuson	-0.63	0.40	>0.999	-2.01	0.76
	Contuson + 6w MNSC vs. Contuson	0.63	0.40	>0.999	-0.76	2.01

Bonferroni multiple comparison. MNSC, Mononuclear Stem Cells.

Table 4 shows that all histological parameters evaluated, both qualitative and quantitative, showed a statistically significant difference between the groups (p < 0.05), except for the cranial group (p = 0.104).

**Table 4:** Description of histological parameters according to groups and results of comparisons.

Variable	Group						p
	Group 1	Group 2	Group 3	Group 4	Group 5	Group 6	
**Necrosis**							<0.001
Absent	8 (100)	8 (100)	0 (0)	0 (0)	0 (0)	8 (100)	
Mild	0 (0)	0 (0)	8 (100)	8 (100)	8 (12.5)	0 (0)	
Moderate	0 (0)	0 (0)	0 (0)	0 (0)	5 (62.5)	0 (0)	
Severe	0 (0)	0 (0)	0 (0)	0 (0)	2 (25)	0 (0)	
**Hemorraghe**							<0.001
Absent	6 (75)	6 (75)	0 (0)	1 (12.5)	0 (0)	6 (75)	
Mild	2 (25)	2 (25)	6 (75)	7 (87.5)	1 (12.5)	2 (25)	
Moderate	0 (0)	0 (0)	2 (25)	0 (0)	7 (87.5)	0 (0)	
Severe	0 (0)	0 (0)	0 (0)	0 (0)	0 (0)	0 (0)	
**Hyperemia**							0.004
Absent	5 (62.5)	5 (62.5)	2 (25)	6 (75)	0 (0)	5 (62.5)	
Mild	3 (37.5)	3 (37.5)	1 (12.5)	0 (0)	3 (37.5)	3 (37.5)	
Moderate	0 (0)	0 (0)	3 (37.5)	2 (25)	4 (50)	0 (0)	
Severe	0 (0)	0 (0)	0 (0)	0 (0)	1 (12.5)	0 (0)	
**Degeneration**							<0.001
Absent	8 (100)	8 (100)	0 (0)	0 (0)	0 (0)	8 (100)	
Mild	0 (0)	0 (0)	5 (62.5)	5 (62.5)	5 (62.5)	0 (0)	
Moderate	0 (0)	0 (0)	3 (37.5)	3 (37.5)	2 (25)	0 (0)	
Severe	0 (0)	0 (0)	0 (0)	0 (0)	0 (0)	0 (0)	
**Infiltrated**							<0.001
Absent	8 (100)	8 (100)	5 (62.5)	7 (87.5)	1 (12.5)	8 (100)	
Mild	0 (0)	0 (0)	1 (12.5)	0 (0)	5 (62.5)	0 (0)	
Moderate	0 (0)	0 (0)	2 (25)	1 (12.5)	2 (25)	0 (0)	
Severe	0 (0)	0 (0)	0 (0)	0 (0)	0 (0)	0 (0)	
**Cranial**							0.104*
Mean ± SD	522.9 ± 114.5	485.5 ± 90.8	412.4 ± 63.6	444.1 ± 63.1	680.6 ± 85.4		
Median (min.; max.)	544 (343; 677	467 (344; 633)	410.5 (332; 533)	404.5 (389; 544)	683.5 (558; 776)		
**Caudal**							<0.001*
Mean ± SD	398.8 ± 56.7	417.4 ± 71.3	239.3 ± 33.9	259.8 ± 18.3	697.9 ± 105		
Median (min.; max.)	422 (312; 467	410 (298; 554)	223.5 (209; 300)	260 (233; 289)	704 (553; 839)		

Teste KruskaI-WaIIis.

⁎ ANOVA; Sham group (Group 6) didn't go for analysis.

**Fig. 3:**
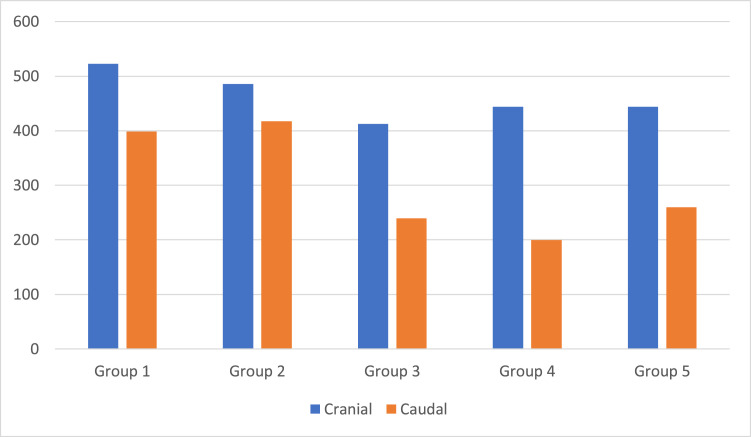
Histological analysis of axonal sprouting.

The Fig. 3 shows the proximal and caudal axonal budding at the lesion site. Groups 1 and 2 showed greater proximal axonal budding, but without statistical significance. The same occurred in the distal axonal budding, however, with statistical significance.

Table 5 shows that, in general, the qualitative histological parameters were statistically better in the groups treated effectively than in the groups treated with saline or untreated solution (p < 0.05), only the cell infiltrate was statistically worse in the lesion group than in all other groups (p < 0.05).

**Table 5:** Results of multiple comparisons between the groups of qualitative histological parameters that differed between the groups.

Variable	Comparison	Z value	p
Necrosis	Contuson + 3w MNSC vs. Contuson + 6w M NSC	0.00	>0.999
	Contuson + 3w MNSC vs. Contuson + 3w saline	-3.74	**<0.001**
	Contuson + 3w MNSC vs. Contuson + 6w saline	-3.74	**<0.001**
	Contuson + 3w MNSC vs. Contuson	-6.11	**<0.001**
	Contuson + 6w MNSC vs. Contuson + 3w saline	-3.74	**<0.001**
	Contuson + 6w MNSC vs. Contuson + 6w saline	-3.74	**<0.001**
	Contuson + 6w MNSC vs. Contuson	-6.11	**<0.001**
	Contuson + 3w MNSC vs. Contuson + 6w saline	0.00	>0.999
	Contuson + 3w MNSC vs. Contuson	-2.38	**0.017**
	Contuson + 6w MNSC vs. Contuson	-2.38	**0.017**
Hemorraghe	Contuson + 3w MNSC vs. Contuson + 6w M NSC	0.00	>0.999
	Contuson + 3w MNSC vs. Contuson + 3w saline	-3.40	**<0.001**
	Contuson + 3w MNSC vs. Contuson + 6w saline	-2.19	**0.028**
	Contuson + 3w MNSC vs. Contuson	-5.31	**<0.001**
	Contuson + 6w MNSC vs. Contuson + 3w saline	-3.40	**<0.001**
	Contuson + 6w MNSC vs. Contuson + 6w saline	-2.91	0.028
	Contuson + 6w MNSC vs. Contuson	-5.31	**<0.001**
	Contuson + 3w MNSC vs. Contuson + 6w saline	1.20	0.228
	Contuson + 3w MNSC vs. Contuson	-1.91	0.056
	Contuson + 6w MNSC vs. Contuson	-3.11	**0.002**
Hyperemia	Contuson + 3w MNSC vs. Contuson + 6w M NSC	0.00	>0.999
	Contuson + 3w MNSC vs. Contuson + 3w saline	-2.87	**0.004**
	Contuson + 3w MNSC vs. Contuson + 6w saline	-0.14	0.887
	Contuson + 3w MNSC vs. Contuson	-3.50	**<0.001**
	Contuson + 6w MNSC vs. Contuson + 3w saline	-2.87	**0.004**
	Contuson + 6w MNSC vs. Contuson + 6w saline	-0.14	0.887
	Contuson + 6w MNSC vs. Contuson	-3.50	**<0.001**
	Contuson + 3w MNSC vs. Contuson + 6w saline	2.73	**0.006**
	Contuson + 3w MNSC vs. Contuson	-0.62	0.533
	Contuson + 6w MNSC vs. Contuson	-3.35	**<0.001**
Degeneration	Contuson + 3w MNSC vs. Contuson + 6w M NSC	0.00	>0.999
	Contuson + 3w MNSC vs. Contuson + 3w saline	-4.49	**<0.001**
	Contuson + 3w MNSC vs. Contuson + 6w saline	-4.49	**<0.001**
	Contuson + 3w MNSC vs. Contuson	-4.62	**<0.001**
	Contuson + 6w MNSC vs. Contuson + 3w saline	-4.49	**<0.001**
	Contuson + 6w MNSC vs. Contuson + 6w saline	-4.49	**<0.001**
	Contuson + 6w MNSC vs. Contuson	-4.62	**<0.001**
	Contuson + 3w MNSC vs. Contuson + 6w saline	0.00	>0.999
	Contuson + 3w MNSC vs. Contuson	-0.13	0.897
	Contuson + 6w MNSC vs. Contuson	-0.13	0.897
Infiltrated	Contuson + 3w MNSC vs. Contuson + 6w M NSC	0.00	>0.999
	Contuson + 3w MNSC vs. Contuson + 3w saline	-1.80	0.072
	Contuson + 3w MNSC vs. Contuson + 6w saline	-0.65	0.514
	Contuson + 3w MNSC vs. Contuson	-3.78	**<0.001**
	Contuson + 6w MNSC vs. Contuson + 3w saline	-1.80	0.072
	Contuson + 6w MNSC vs. Contuson + 6w saline	-0.65	0.514
	Contuson + 6w MNSC vs. Contuson	-3.78	**<0.001**
	Contuson + 3w MNSC vs. Contuson + 6w saline	1.15	0.252
	Contuson + 3w MNSC vs. Contuson	-1.98	**0.048**
	Contuson + 6w MNSC vs. Contuson	-3.13	**0.002**

Dunn's multiple comparisons test.

The flow parameter showed a higher mean value in the effectively treated groups than in the other groups (p < 0.001). The caudal parameter of axonal sprouting (Table 6) presented a statistically higher average value in the effectively treated groups than in the other groups (*p* < 0.001).

**Table 6:** Result of multiple comparisons between the groups of caudal histological parameters.

Caudal					
Comparison	Mean difference	Standard Error	p	95 % CI	Mean difference
Contuson + 3w MNSC vs. Contuson + 6w M NSC	-18.6	25.4	>0.999	-94.6	57.4
Contuson + 3w MNSC vs. Contuson + 3w saline	159.5	25.4	**<0.001**	83.5	235.5
Contuson + 3w MNSC vs. Contuson + 6w saline	199.4	25.4	**<0.001**	123.4	275.4
Contuson + 3w MNSC vs. Contuson	139.0	25.4	**<0.001**	63.0	215.0
Contuson + 6w MNSC vs. Contuson + 3w saline	178.1	25.4	**<0.001**	102.1	254.1
Contuson + 6w MNSC vs. Contuson + 6w saline	218.0	25.4	**<0.001**	142.0	294.0
Contuson + 6w MNSC vs. Contuson	157.6	25.4	**<0.001**	81.6	233.6
Contuson + 3w MNSC vs. Contuson + 6w saline	39.9	25.4	>0.999	-36.1	115.9
Contuson + 3w MNSC vs. Contuson	-20.5	25.4	>0.999	-96.5	55.5
Contuson + 6w MNSC vs. Contuson	-60.4	25.4	0.229	-136.4	15.6

Bonferroni multiple comparison. MNSC, Mononuclear Stem Cells.

## Discussion

Injuries to the central nervous system in mammals, a group that includes humans, were thought to be incapable of regeneration or repair. However, it was seen that the tissue has the capacity to perform some type of repair or regeneration. Restoration, even if limited, can generate great benefits for spinal cord injured patients.^20^^,^^21^

The use of stem cells in individuals with neurological injuries is an important front of study for the rehabilitation of neurological injuries. The role of stem cells in spinal cord injured patients is to modulate the immune response to decrease inflammation at the site of injury, release nutritional factors to promote neurological recovery and stimulate angiogenesis to remodel the blood-brain barrier.^22^

The extracellular matrix, such as metalloproteinases, play a fundamental role in the neuronal recovery environment.^23^ Mesenchymal stem cells play an important role in its regulation by controlling its deposition and degradation. They also exert a paracrine effect through the release of extracellular vesicles that contain various bioactive molecules such as proteins, nucleic acids and lipids.^24^ These vesicles can transfer their contents to cells at the site of spinal cord injury, thus contributing to their gene expression, proliferation and differentiation, resulting in better neuronal recovery.^25^

In a study published in 2017 in rats, it was questioned which would be the best time elapsed from the injury for the injection of stem cells in the spinal cord injury. Injection of human neural stem cells immediately after injury, 7 days after initial injury, and 28 days later were tested. All groups had improvement in their motor function, with the subacute injection group (7 days) having the best response.^6^ In a meta-analysis, it was also found that the best period for experimental treatment in rats with stem cells was the subacute.^7^ However, this meta-analysis did not include studies of stem cell injection in the chronic phase, limiting the studies selected to the injection period from 0 to 10 days after spinal cord injury. In addition, questions of methodological differences must be raised that may weigh on the comparison between the cited works and the present study. The aforementioned studies are carried out in rats, the route of implantation of stem cells is not unified among them, in addition to the number of cells injected and the cell type being different, being neuronal stem cells.

Due to the gap in the literature, due to heterogeneous studies of different quality that still do not provide enough information, the present study sought to help answer whether the treatment with stem cells in mice in two different periods of the chronic phase, with 3 and 6 weeks of injury, would bring therapeutic benefit to the studied animals.

In another study carried out in rats, stem cells were injected at the site of spinal cord injury at 7 days and 10 months. The first group showed motor improvement compared to controls and histological improvement of remyelination and greater neuronal connection. The 10-month group showed that the stem cells were still viable at the injury site when the animals were sacrificed, however the animals did not show motor or histological improvement in relation to the controls. It so happens that, in the chronic phase, the extensive astrogliosis showed that the axons were gradually being phagocytosed by the astrocytes, preventing new connections from being made by the stem cells with the possible axons available.^8^

In work carried out with mice, with stem cells and neural precursors, the placement of stem cells in the spine after spinal cord injury was compared, at the time of injury (acute), 7 days (sub-acute) and 3 months after (chronic). All groups showed an increase in regenerative cells and neutrophils at the injury site, with chronic cases allowing the differentiation of stem cells into neurons and oligodendrocytes, with a more neurogenic environment than the other groups. The acute and subacute groups showed motor improvement on the BMS scale in relation to the control, different from the group with cell implantation in the chronic phase.^9^

When analyzing Fig. 1, it can be seen that there was partial neurological improvement over the 12 weeks in the groups. The groups with stem cell injection at 3 weeks and 6 weeks (groups 1 and 2) showed the greatest neurological recovery. In the statistical analysis, a statistically significant difference was seen within the groups at different times, with the exception between 6 and 12 weeks of injury in groups 3, 4 and 5. Comparing the different groups in relation to time, a statistical difference was found between the groups with stem cell injection at the 12^th^ week, except when comparing groups 1 and 2 and groups 2 and 4.

The present study reached the result of a better motor response in the animals that were implanted with stem cells closer to the subacute phase, being consistent with the results found in the current literature, although there was no statistically significant difference between the results of Group 1 and Group 2. By showing motor improvement in the chronic phase, the present study suggests that there may be a time gap in the chronic phase where it is still feasible to transplant stem cells at the injury site, where there is not so much astrogliosis and there are still viable axons for new connections nerves and more possibilities for neuroplasticity.^8^

The analysis of Fig. 2 together with Table 3 demonstrates better neurological recovery of the groups that underwent stem cell implantation in relation to the other groups, especially when comparing the groups with 12 weeks of injury. When comparing the results obtained by the BMS and MFS scales, despite not being identical, they are quite similar, which is consistent with analyzes carried out in the medical literature.^26^, ^27^, ^28^ Ung et al.^26^ compared the Basso, Beattie and Bresnahan (BBB), Basso Mouse Score (BMS), Antri, Orsal and Barthe (AOB), Motor Function Score (MFS) and Averaged Combined Score (ACOS) in mice with spinal cord injury and evaluated their spontaneous recovery up to 5 weeks. With the exception of ACOS, all obtained similar results.

The two scales used, BMS and MFS, are widely used for functional assessment, specific for mice, and show excellent inter- and intra-observer agreement.^16^^,^^17^ In another study carried out in the laboratory, Torelli et al.^27^ studied the effect of GM1 gangliosides and erythropoietin in mice with spinal cord injury, and as well as the study by Ung et al.,^26^ the results of the BMS and MFS scales were similar, which is consistent with what the authors found in this study.

After observing the mice for 12 weeks, they were sacrificed for histopathological analysis. Spinal cord injury, from the histological point of view, presents mechanical destruction that leads to hemorrhage, decreased perfusion, tissue hypoxia, edema and, finally, necrosis of neural elements. In the groups studied in this work, the findings showed some degree of morphological alteration depending on the treatment instituted, which is consistent with the findings in the medical literature.^29^

Among the intervention groups, a better histopathological result was found in all aspects analyzed, such as necrosis, hemorrhage, cellular infiltrate, hyperemia and degeneration, with statistical significance. In addition, distal axonal sprouting was greater in the intervention groups than in the other groups, with statistical significance, and the proximal axonal sprouting tended to be greater, however without statistical significance.

In a recent study, implantation of mesenchymal cells was performed on the day of the injury associated with physical training on a treadmill with the mice being followed up for 8 weeks. Histological analysis found mild infiltration of inflammatory cells in the injured tissue. In the control group (injury), severe infiltration of inflammatory cells was found.^30^ The results are similar to those of the present study; however, they are not identical. This difference can be explained by the difference in cell volume (1 × 10^5^ vs. 4 × 10^5^), cell origin (bone marrow × umbilical cord), time from injury to injection (immediate × 3 and 6 weeks) and time to sacrifice the animal (8 weeks × 12 weeks).

Other studies, with different methods, point to similar results of neuronal recovery from the histological point of view. Ai Takahashi et al.,^31^ in a study carried out with rats and with mesenchymal cells from adipose tissue, found histological improvement with stem cell transplantation associated or not with exercise, with less formation of bedsore and cavity at the injury site 4 weeks after the trauma. Despite the histological improvement occurring with or without exercise, motor improvement only occurred in rats that underwent an exercise program. Such clinical-histological discrepancy is compatible with real-life situations in humans, and such situations frequently occur in humans. doctors' office. Son et al.,^32^ in a study carried out in rats with stem cells created from genetic engineering (induced pluripotent stem cells), observed histological improvement by a method other than HE, associated with motor improvement in the studied rats, and the stem cell transplant was performed immediately after the injury and the animals were sacrificed 8 weeks after the spinal cord injury. Another data that is consistent with the current study is that there was an increase in axonal sprouting both proximally and distally.

This study presents relevant results for studies on the use of stem cells in spinal cord injured patients, thus contributing to bring us closer and closer to the clinical use that is fully available to the needy population. There are other strengths in this study: it's a laboratory-controlled study with a good study design for what it was meant for with very little risk of bias that had interfered in the result. Despite the important outcomes, the study also has flaws. It would have provided more data and would have been more impactful if the study had carried out analyzes of imaging tests such as magnetic resonance imaging, had performed immunohistochemistry to assess cell composition and the expression of receptors at the site of injury. An electroneuromyography could also have been performed at the end of the study to analyze the neuromotor recovery. Other limitations of the study are the small sample and the limitation of post-injury time evaluation. If it had a bigger sample probably and a wider time in post-injury evaluation It would have more statistically significant results. At the end of these considerations, it is seen that despite the flaws, the present study brings important and unprecedented information to the scientific literature on the result of the implantation of stem cells in mice in different periods of the chronic phase, thus contributing to the scientific advancement and use stem cells in clinical practice.

## Conclusion

The present study demonstrated that the implantation in mice of mononuclear stem cells obtained in human umbilical cord and placental blood at the injury site, 3 to 6 weeks after spinal cord injury, improves their motor and histopathological response.

## CRediT authorship contribution statement

**Thiego Pedro Freitas Araujo:** Conceptualization, Data curation, Formal analysis, Investigation, Methodology, Writing – original draft. **Alexandre Fogaça Cristante:** Conceptualization, Methodology, Project administration, Supervision, Writing – review & editing. **Raphael Martus Marcon:** Conceptualization. **Gustavo Bispo dos Santos:** Investigation, Methodology. **Maria Helena Alves Nicola:** Conceptualization. **Alex Oliveira de Araujo:** Investigation, Methodology. **Fernando Barbosa Sanchez:** Investigation, Methodology. **Tarcísio Eloy Pessoa de Barros Filho:** Supervision, Validation, Visualization.

## Declaration of competing interest

The authors declare no conflicts of interest.
